# Reframing Sustainable and Planetary Health Through the Fundamentals of Care: A Conceptual Nursing Perspective

**DOI:** 10.1111/nin.70095

**Published:** 2026-03-19

**Authors:** Susana Isabel Rodrigues de Sul, Paulo Jorge Silva Nogueira, Andreia Cátia Jorge Silva Costa

**Affiliations:** ^1^ Nursing Research Innovation and Development Centre of Lisbon (CIDNUR), School of Nursing Universidade de Lisboa Lisbon Portugal; ^2^ Institute of Environmental Health (ISAMB), Faculty of Medicine Universidade de Lisboa Lisbon Portugal; ^3^ Laboratory for the Sustainability of Land Use and Ecosystem Services (TERRA) Lisbon Portugal

**Keywords:** Fundamentals of Care, health literacy, nursing theory, planetary health, sustainable health

## Abstract

This discursive conceptual paper reframes sustainable and planetary health through a nursing‐centred lens by applying the Fundamentals of Care framework to contemporary challenges including population ageing, multimorbidity, climate disruption, digital fragmentation and widening social inequities. It argues that the Fundamentals of Care provide an integrative framework capable of connecting the physical, psychosocial and relational dimensions of care with broader agendas of sustainable and planetary health. By positioning nursing practice at the interface where macro‐level societal pressures are translated into lived experience, the framework illuminates how health literacy, digital literacy, Healthy Cities and self‐care in ageing can be understood as relationally and environmentally constituted capabilities rather than as individual responsibilities alone. From this perspective, health and digital literacy emerge as strategic public capabilities that strengthen resilience to misinformation, polarisation and civic disengagement, while Healthy Cities illustrate how environmental design and governance can promote well‐being and equity at scale. Attention to political, commercial and digital determinants further highlights the need for relational infrastructures that foster trust, participation and inclusion. The paper concludes that the Fundamentals of Care positions nursing practice as a relational infrastructure that links individuals, communities and systems, translating sustainability agendas into lived experience and enabling sustainable and planetary health to advance in ways that connect policy ambitions with equity and long‐term societal resilience.

## 1 Introduction: Towards a New Understanding of Health Investment

Health systems are undergoing a profound transition. Ageing populations, rising multimorbidity and chronic disease are reshaping the organisation and delivery of care (WHO 2015; Organisation for Economic Co‐Operation and Development 2021). Climate change is altering environmental exposures, increasing heat‐related mortality and exacerbating respiratory and cardiovascular conditions (Romanello et al. 2024). Digital ecosystems have become essential infrastructure for governance and health communication, yet also serve as vectors for misinformation (Okan and Marchandise 2025). Rapid urbanisation further concentrates environmental risks and social inequities, while also creating opportunities for health‐promoting design and collective action (Cacciatore et al. 2025).

The concept of planetary health, initially articulated by the Rockefeller Foundation–Lancet Commission, foregrounds the interdependence between human health and the integrity of natural systems (Whitmee et al. 2015). It emphasises that population well‐being cannot be sustained independently of ecological stability, climate regulation and planetary boundaries. Closely related, sustainable health is understood in alignment with the United Nations 2030 Agenda for Sustainable Development as the long‐term capacity of societies and health systems to promote well‐being without compromising the environmental, social and institutional foundations on which present and future generations depend (United Nations [UN] 2015). While sustainable and planetary health have gained considerable prominence in policy and global health discourse, they have largely been framed at macro‐ecological and governance levels, with less attention to how these interdependencies are enacted within everyday relational care practices.

These forces are contributing to a redefinition of what it means to invest in health. Across Europe, policy debates reflect this shift. The *Eurohealth* Special Issue on *Investing for Sustainable Health and Well‐Being* and the theme of the 18th European Public Health Conference both emphasise that health must be understood as a long‐term investment in social, economic and democratic resilience (European Observatory on Health Systems and Policies [EOHSP] 2025). Investments in sustainable health can therefore extend beyond healthcare to encompass literacy, environments, trust, governance and social infrastructures that support participatory and cohesive societies. It also resonates strongly with the United Nations Sustainable Development Goals (SDGs), especially SDG 3 (Good Health and Well‐Being), SDG 10 (Reduced Inequalities), SDG 11 (Sustainable Cities and Communities) and SDG 13 (Climate Action), all of which underscore the interdependence between planetary stability, social cohesion and population health (UN 2015).

Nursing scholarship has increasingly engaged with sustainable and planetary health through work on climate‐responsive care, environmental stewardship, sustainable healthcare systems and professional advocacy (e.g., Kurth 2017; Butterfield et al. 2021). Much of this literature has emphasised the environmental footprint of healthcare and the ethical responsibilities of the profession. Less attention, however, has been given to how these agendas are operationalised within everyday relational care encounters. This gap invites conceptual frameworks capable of linking macro‐level ecological concerns with micro‐level practices of care.

Despite growing attention to sustainable and planetary health at macro‐policy levels, much less is known about how these agendas translate into everyday encounters where people make sense of information, navigate vulnerability and build capacity for action (Romanello et al. 2024; EOHSP 2025). Addressing this gap requires conceptual frameworks capable of integrating the physical, psychosocial and relational dimensions through which health is enacted in daily life.

Nursing practice occupies a distinctive position at this interface. Nurses work where societal pressures—such as climate‐related risks, digital misinformation, chronic illness and social isolation—are experienced as lived, relational and psychosocial challenges (A. L. Kitson 2018; A. L. Kitson et al. 2023). Through continuity of care, relational proximity and trust, nursing practice offers a unique lens for understanding how sustainable and planetary health can be operationalised beyond abstract policy aspirations.

This discursive paper advances a nursing‐centred conceptualisation of sustainable and planetary health by arguing that contemporary challenges require integrative models of practice capable of uniting relational, psychosocial and physical dimensions of care. The argument is developed through three illustrative exemplars: (1) health and digital literacy; (2) Healthy Cities; and (3) self‐care in ageing, selected to demonstrate the analytic utility of the Fundamentals of Care (FoC) framework. Kitson's FoC provide the conceptual lens guiding this analysis by specifying how health creation emerges from dynamic interactions between people, systems and environments (A. L. Kitson 2018; A. L. Kitson et al. 2023). The central argument is that sustainable and planetary health depend on relational infrastructures enacted through everyday nursing practice, enabling individuals and communities to engage with information, environments and institutions in ways that support equitable and long‐term societal resilience.

## 2 Health Literacy and Digital Literacy as Strategic Public Health Investments

Health and digital literacy are emerging as central determinants of resilience within complex information ecosystems (Galea et al. 2025; UNESCO 2018; Sørensen et al. 2012). As individuals navigate medical uncertainty, algorithmically curated content and shifting scientific guidance, literacy has become a form of civic competence. Low health literacy has been linked to vaccine hesitancy, delayed care‐seeking and susceptibility to conspiracy theories, particularly during the COVID‐19 infodemic (Okan and Marchandise 2025; Loomba et al. 2021; Cinelli et al. 2020).

Digital literacy intersects with these challenges by shaping how individuals interpret risk, assess sources and recognise manipulative content. Commercial digital platforms have structural incentives to amplify sensational and emotionally charged material, increasing exposure to misinformation and undermining trust in public institutions (Okan and Marchandise 2025; Cinelli et al. 2020). In this context, strengthening literacy can operate as a preventive intervention against political polarisation and democratic erosion. Its broader societal relevance has led some scholars to characterise it as a ‘social vaccine’ (Okan and Marchandise 2025).

The FoC helps to conceptualise why literacy‐building efforts are more likely to succeed when anchored in relationships. Traditional literacy interventions often emphasise cognitive skills—reading, interpreting and evaluating—but evidence demonstrates that such skills become actionable only when individuals feel respected, supported and emotionally safe. FoC highlights that effective communication and comprehension require relational trust, psychosocial support and attention to physical needs. When individuals experience fear, shame or disconnection, even high‐quality information may fail to translate into behaviour (A. L. Kitson 2018). Conversely, when they feel heard, valued and supported, information becomes a lived capability rather than an overwhelming cognitive task (Nielsen et al. 2024; Weerakoon et al. 2022).

Nurses play a key role in operationalising literacy‐building strategies. Their relational proximity enables them to contextualise information, address fears and misconceptions and reinforce confidence through dialogue. The FoC illuminate why literacy initiatives succeed when integrated with relational trust, psychological safety and attention to physical needs. When individuals feel supported, respected and understood, information becomes actionable rather than overwhelming. Literacy thus becomes a lived and socially embedded capability rather than a purely cognitive task (A. L. Kitson et al. 2023; Nielsen et al. 2024; Weerakoon et al. 2022).

Improving health and digital literacy is also a direct contribution to the SDGs, particularly SDG 4.7 (education for sustainable development) and SDG 16 (peace, justice and strong institutions), both of which prioritise informed participation, trust‐building and societal resilience. Strengthening literacy through nursing‐led initiatives therefore operates simultaneously as a public health and democratic investment (UN 2015).

From the perspective of advanced nursing practice, these findings highlight that health and digital literacy should be recognised as core components of sustainable care rather than ancillary educational activities. Evidence shows that literacy becomes actionable when supported by relational continuity, trust and psychosocial safety (Nielsen et al. 2024; Weerakoon et al. 2022). Through the FoC lens, advanced nurses are particularly well positioned to integrate literacy support into routine encounters by contextualising information, addressing uncertainty and supporting sense‐making (A. L. Kitson 2018; A. L. Kitson et al. 2023). Strengthening literacy through relational nursing practice therefore represents a concrete contribution to resilience, equity and democratic participation, particularly in contexts marked by misinformation and digital fragmentation (Okan and Marchandise 2025; Sørensen et al. 2012; Cinelli et al. 2020; Loomba et al. 2021).

## 3 Healthy Cities as Ecosystems for Sustainable Health

Urban environments are key ecosystems in shaping population health, mediating both exposure to environmental risks and access to health‐promoting resources. While cities frequently concentrate air pollution, traffic, noise, heat islands and sedentary lifestyles, they also offer opportunities for physical activity, social interaction, cultural participation and access to essential services (World Health Organization, & Metropolis 2014). Within Healthy Cities frameworks, health is understood as an emergent property of complex urban systems, produced not only by the health sector but by transport, housing, energy, mobility and public space governance. When urban planning prioritises clean air, active and safe mobility, walkable neighbourhoods, green infrastructure and equitable access to public services, it creates enabling environments that enhance physical and mental well‐being while mitigating social and health inequities (World Health Organization, & UN‐Habitat 2016).

Over the last decade, numerous cities—including Barcelona, Copenhagen, New York, Paris and Singapore —have demonstrated that strategic redesigns of streets, mobility networks and public space are associated with simultaneous environmental and health gains. Improvements in air quality, expansion of green areas, pedestrian‐ and cyclist‐oriented infrastructures and the development of inclusive public spaces have contributed not only to reductions in exposure to risk factors but also to strengthened social cohesion and overall quality of life. These cases underscore that urban design can operate as an important health policy instrument, capable of influencing population health at scale (World Health Organization, & Metropolis 2014; Pérez et al. 2025).

Barcelona's Superblocks model is one of the most thoroughly documented examples of health‐centred urban planning. By reorganising groups of city blocks to restrict through‐traffic, calm internal circulation and reclaim streets for pedestrians, cyclists and community uses, Superblocks transform car‐dominated areas into open, socially vibrant and environmentally restorative urban micro‐ecosystems. Empirical assessments show consistent improvements in environmental indicators—such as reductions of up to 25% in NO₂ and notable decreases in particulate matter and noise levels—as well as behavioural shifts towards increased walking, cycling and social interaction. A health‐impact assessment estimated that full implementation of the city's planned 503 Superblocks could prevent around 667 premature deaths annually and increase life expectancy by approximately 200 days, illustrating the significant potential of urban design interventions for population health promotion (Pérez et al. 2025).

From a FoC perspective, these urban transformations are significant not only because they reduce emissions or pollution, but because they reshape the relational and psychosocial conditions under which care is experienced. Walkable neighbourhoods, accessible green spaces and safe public infrastructures alter the physical preconditions for mobility, interaction and participation, particularly for older adults and people living with chronic illness. FoC draws attention to how environmental design influences relational continuity, dignity and the capacity to engage in everyday self‐care, thereby translating urban policy into lived health capability.

Healthy Cities frameworks increasingly incorporate psychosocial well‐being and community resilience. Loneliness and social isolation, particularly among older adults, have emerged as critical public health challenges (Holt‐Lunstad 2022). Urban environments that support walkable neighbourhoods, proximate community resources and intergenerational encounters function as protective structures. In this context, nursing professionals—especially in primary care and community settings—act as intermediaries, helping residents navigate environmental health risks, access local assets and participate in community networks. Their holistic perspective and proximity to vulnerable groups position them as key advocates for urban policies attentive to ageing, disability, chronic disease and socioeconomic disadvantage (Alanazi et al. 2024).

Finally, Healthy Cities align closely with sustainable and planetary health principles. Investments in active mobility, green infrastructure and climate‐resilient design simultaneously reduce greenhouse gas emissions, enhance biodiversity and promote human well‐being. These co‐benefits reinforce the notion that sustainable health depends on cross‐sectoral governance and long‐term, systems‐level perspectives capable of integrating environmental, social and health objectives. In this sense, building Healthy Cities is not only an urban agenda but a public health imperative within a changing climate (EOHSP 2025). Urban health promotion is directly linked to the SDGs, especially SDG 11 on Sustainable Cities and SDG 13 on Climate Action. Healthy Cities therefore exemplify how public health, environmental policy and social cohesion can be integrated within a single transformative agenda (UN 2015).

## 4 Beyond Individual Responsibility: Rethinking Self‐Care in Ageing

Healthy ageing frameworks emphasise functional ability and autonomy rather than disease absence. As multimorbidity becomes the norm in older populations, self‐care practices—ranging from medication management to physical activity and symptom monitoring—are essential for maintaining independence and reducing avoidable health system use (WHO 2015).

However, self‐care is often framed narrowly as an individual responsibility. In practice, it is shaped by relational continuity, emotional support, digital access, environmental design and the broader social determinants of health. Digital health technologies offer new opportunities for monitoring and engagement, yet they risk excluding those with limited digital literacy, sensory impairments or precarious socioeconomic conditions (Organisation for Economic Co‐Operation and Development 2021). Without relational and psychosocial support, technology‐mediated self‐care remains inaccessible to many older adults.

Nursing practice plays an important role in enabling meaningful self‐care. Nurses help individuals interpret bodily sensations, navigate uncertainty, build routines and develop confidence in decision‐making. Through an FoC lens, self‐care support must address physical needs, psychosocial factors and relational trust simultaneously. Older adults who feel heard and respected are more likely to adopt new technologies, engage with community resources and maintain autonomy. This relational and psychosocial support is a key contributor to the sustainability of self‐care (Organisation for Economic Co‐Operation and Development 2021; A. L. Kitson 2018; Beaudin et al. 2024).

The political discourse surrounding self‐care also requires reframing. Framed narrowly as personal responsibility, self‐care risks reinforcing inequities by obscuring the environmental, social and digital barriers that constrain individual agency. A FoC‐informed approach acknowledges these constraints and recognises that self‐care is co‐produced within relationships, communities and institutions (A. L. Kitson 2018; A. Kitson et al. 2025).

For advanced nursing practice, this reframing of self‐care has important implications. Supporting sustainable self‐care in ageing populations requires more than promoting individual responsibility; it depends on relational continuity, psychosocial support and attention to environmental and digital barriers (WHO 2015; Organisation for Economic Co‐Operation and Development 2021; Beaudin et al. 2024). The FoC framework clarifies how advanced nurses can enable sustainable self‐care by fostering trust, supporting interpretation of bodily signals and adapting care to individuals' functional, emotional and social contexts (A. L. Kitson et al. 2023; A. Kitson et al. 2025). Such relationally grounded approaches are essential to ensuring that self‐care initiatives contribute to equity and sustainability rather than reinforcing existing disparities (A. L. Kitson 2018; Beaudin et al. 2024).

## 5 The Expanding Influence of Political, Commercial and Digital Determinants

Sustainable health cannot be achieved without addressing broader structural determinants. Commercial actors shape consumer environments through marketing, pricing strategies and digital advertising infrastructures that influence behaviour and belief formation, while political dynamics—including austerity measures, populism and weakened public institutions—affect trust, participation and the equitable distribution of resources (Galea et al. 2025). From a FoC perspective, these macro‐level forces condition the relational climate within which care is delivered and experienced, influencing whether encounters foster a sense of safety, recognition and meaningful engagement.

Individuals with poorer health, low literacy or precarious socioeconomic conditions are disproportionately exposed to manipulative digital content and targeted advertising. Evidence suggests that these groups often experience lower institutional trust and higher susceptibility to simplified or polarising narratives, creating feedback loops that reinforce disadvantage and undermine democratic resilience (Okan and Marchandise 2025; Galea et al. 2025). These dynamics highlight the interconnections between health, democracy and digital ecosystems.

Nursing leadership constitutes an important interface between structural determinants and population well‐being. Positioned in close contact with communities, nurses are well placed to detect how commercial practices, digital platforms and political dynamics translate into everyday barriers to health. This proximity allows them to advocate for regulatory and governance measures that curb harmful digital marketing, promote transparent information ecosystems and strengthen institutional trust. Moreover, their high relational credibility enables nurses to act as stabilising agents in contexts marked by misinformation, political polarisation and declining civic engagement. By fostering digital literacy, supporting inclusive participation and promoting equitable access to health‐promoting environments, both physical and digital, nursing leadership contributes to counteracting the feedback loops that perpetuate socioeconomic disadvantage and erode democratic resilience (Alanazi et al. 2024).

From a leadership perspective, this positions advanced nursing not only as a clinical role but as a stabilising social actor within fragmented information and governance environments. By combining relational credibility with advocacy competencies, nursing leadership can contribute to policy debates addressing commercial, political and digital determinants of health, particularly those shaping exposure to misinformation and institutional trust (Galea et al. 2025; Okan and Marchandise 2025; Alanazi et al. 2024).

## 6 Synthesis: From Macro‐Level Agendas to Everyday Care Practices

Contemporary health challenges extend far beyond the boundaries of traditional healthcare systems. The conditions shaping well‐being increasingly span ecological stability, social cohesion, digital infrastructures and civic life, reinforcing the understanding of health as a planetary and societal phenomenon (EOHSP 2025). In this context, sustainable health depends not only on policy and system‐level interventions, but on how individuals and communities are supported to navigate complexity, uncertainty and vulnerability in everyday life.

Socioecological and systems models have significantly advanced the understanding of multilevel determinants of health. However, they primarily map levels of influence rather than specifying the relational mechanisms through which these determinants become embodied in lived experience. The FoC framework complements these approaches by explicating how trust, dignity, communication and attention to physical and emotional needs interact within real‐time care encounters, thereby translating macro‐level sustainability agendas into practice‐level enactment (A. L. Kitson et al. 2023; A. Kitson et al. 2025).

Positioning nursing as central within this expanded vision of health may appear paradoxical given the breadth of planetary challenges. However, this paradox dissolves when considering nurses' unique relational proximity to lived experience. Nurses work at the interface where macro‐level pressures—such as climate‐related risks, misinformation, chronic illness and social isolation—are encountered as personal and relational challenges (A. Kitson et al. 2025; Nielsen et al. 2024; Weerakoon et al. 2022; A. L. Kitson 2018). Through continuity, trust and contextualised support, nursing practice functions as a form of relational infrastructure linking individuals, communities and systems.

For nursing science, this synthesis reinforces the relevance of relational care as a core contribution to contemporary challenges including sustainability, ageing and health equity. For advanced nursing practice and education, it highlights the importance of integrating literacy support, relational continuity and advocacy competencies alongside clinical expertise. Taken together, the FoC framework positions nursing as a key contributor to sustainable and planetary health by connecting everyday care encounters with broader goals of equity, resilience and a liveable future.

## Author Contributions

S.I.R.d.S., P.J.S.N. and A.C.J.S.C. contributed to the conception and design of the article. S.I.R.d.S. wrote the first draft of the manuscript. P.J.S.N. and A.C.J.S.C. revised and added new statements and data to the manuscript. All authors contributed to manuscript revision, read and approved the submitted version.

## Ethics Statement

This article is a perspective and does not involve human participants, human data or animals. Therefore, ethical approval and informed consent were not required.

## Conflicts of Interest

The authors declare no conflicts of interest.

## Data Availability

No datasets were generated or analysed during the current study. Data sharing is not applicable to this article.
